# Supervision of students in a Portuguese veterinary medicine school—students’ and supervisors’ expectations, perceptions, and supervision impact

**DOI:** 10.3389/fvets.2024.1502981

**Published:** 2024-12-24

**Authors:** João Cota, Eva Cunha, Ricardo Bexiga, Manuela Oliveira

**Affiliations:** ^1^CIISA—Centre for Interdisciplinary Research in Animal Health, Faculty of Veterinary Medicine, University of Lisbon, Lisbon, Portugal; ^2^AL4AnimalS—Associate Laboratory for Animal and Veterinary Sciences, Lisbon, Portugal; ^3^3cE3c—Centre for Ecology, Evolution and Environmental Changes and CHANGE—Global Change and Sustainability Institute, Lisbon, Portugal

**Keywords:** veterinary medicine, students, supervision, supervisors, education, expectations

## Abstract

**Introduction:**

The student-supervisor relationship can be a major cause of psychological distress experienced by students during their study programs. Misalignment between students’ and supervisors’ expectations and perceptions can originate conflicts, highly affecting the wellbeing of students and hindering the progress of their studies. This study was based on a questionnaire focusing on the perceptions of students and supervisors regarding the most important student attributes and outcomes, supervision expectations and perceptions, and supervision impact on mental health of students from a Portuguese Veterinary Medicine School.

**Methods:**

The participants included 36 students from different study programs (bachelor’s, master’s, doctoral and veterinary specialization) and their corresponding supervisors (three).

**Results:**

The results indicate that veterinary post-graduate and specialization students and their supervisors share similar expectations regarding key student traits and supervision outcomes. Students expected supervisors to be actively involved, particularly in developing technical skills, and generally rated the supervision as of high or very high quality, emphasizing the importance of supervisors’ commitment. In contrast, supervisors were more focused on fostering students’ autonomy, and their assessments of the supervision quality were more diverse. The factors that most affected student’s mental health were the progress of the research or training program and students’ personal expectations, while supervisors perceived that their expectations also induced a negative impact on students’ mental health.

**Discussion:**

Further and continuous research is needed to better understand both the needs and expectations of students and supervisors in different academic realities, including in Veterinary Medicine schools, from which the information available on the subject is scarce.

## 1 Introduction

Among other outputs or tasks, higher education institutions are expected to provide their students high-level qualifications in all study programs offered (bachelor’s, master’s, and doctoral), to deliver technical/professional competences, and to generate and disseminate knowledge. Either during undergraduate research projects, master’s or doctoral studies, or during technical specialty training programs, students are guided by, at least, one supervisor, a relationship that has been the focus of many studies in past years, mainly concerning doctoral study programs. Supervision is a complex process that is influenced by multiple factors, including the personalities of both the supervisee and the supervisor (1).

When starting a supervised study program, both the student and the supervisor have their own expectations and perceptions, that should be discussed in the beginning of the program (2). Mismatches in expectations, as well as conflicting ways of thinking and working or clashes of personality, can affect the supervision process (3). Additionally, the perceptions on quality and quantity of supervision of both students and supervisors should be aligned to ensure the progress of studies, increase student satisfaction and reduce the risk of study discontinuation (4).

Mental health issues among postgraduate research students has caused academics and higher education institutions to start rethinking the classic method of supervision. Doctoral students are known to experience psychological distress at high levels during the development of their research programs, including anxiety and depression, which can arise from multiple reasons, including the supervision process experienced (5, 6). There are several studies on the mental health and wellbeing of undergraduate and graduate students, which should prompt higher education institutions to act accordingly, but the information available with focus on students from Veterinary Medicine schools is scarce.

Though extensively studied in other institutions and in other countries, previously reported findings on student-supervisor relationships might not be fully transferrable to all academic realities, due to differences in workload, course design, job market, and work-life balance, among others. Moreover, understanding students’ beliefs and insecurities is crucial for supervisors to effectively fulfill their role. Therefore, this research aimed to characterize the expectations, perceptions and the impact of supervision through the eyes of students and supervisors in a Portuguese Veterinary Medicine School.

## 2 Materials and methods

### 2.1 Survey design

An adaption of the survey used by Cardilini et al. (2) was used in the present study. The survey was composed by 12 questions divided in two sections: the first focused on participants’ expectations and the second focused mainly on the impact of the supervision on the students’ mental health. The same survey was used both for students as well as for supervisors.

The first section of the survey had a total of four closed questions with a predefined list of possible answers. The participants were initially asked to select, in their view, which were the five most important student attributes at the beginning and at the end of the supervised training period. In the next question, the participants were requested to select the five most relevant attributes to be developed by students and to which supervisors should contribute the most. Within the framework of this study, attributes are considered to be student features. Finally, the participants were asked to select, from a predefined scale including four hypotheses (none; only when asked; when the supervisor considers it necessary; whenever the opportunity arises), which was the level of guidance sought/provided from/by the supervisor.

The second section of the survey totaled eight Likert scale questions with a five-point scale answer. The first six questions regarded the impact of the experiences lived through the research/training period, the relationship with the supervisor, the environment of the research group, the progress of the research project, the individual expectations and the expectations of the supervisor on the students’ mental health, either from the students’ or from the supervisors’ point of view. The five-point scale answers for these questions ranged from strongly disagree to strongly agree. The two final questions intended to understand the students’ perception of the quality of the supervision and the likelihood of pursuing an academic career. The same two questions were presented to the supervisors, to obtain a self-evaluation of the quality of guidance provided and to assess the supervisors’ expectations on their students’ potentially pursuing an academic career. The five-point scale answers for these questions ranged from very low to very high.

### 2.2 Survey participants

Thirty-six students from different bachelor’s (3/36), master’s (25/36), doctoral (5/36), and veterinary specialization (3/36) study programs, carrying out research activities/training in the Faculty of Veterinary Medicine of the University of Lisbon during the year of 2023, and their supervisors (three), were invited and completed the web-based survey between April and May.

### 2.3 Data analysis

All the answers from the participants were gathered and their identity remained anonymous. Descriptive statistics and graphs were performed using Microsoft Excel (Microsoft Corporation, Redmond, WA, USA).

## 3 Results

### 3.1 Students’ attributes and supervisor guidance

**Figure 1:**
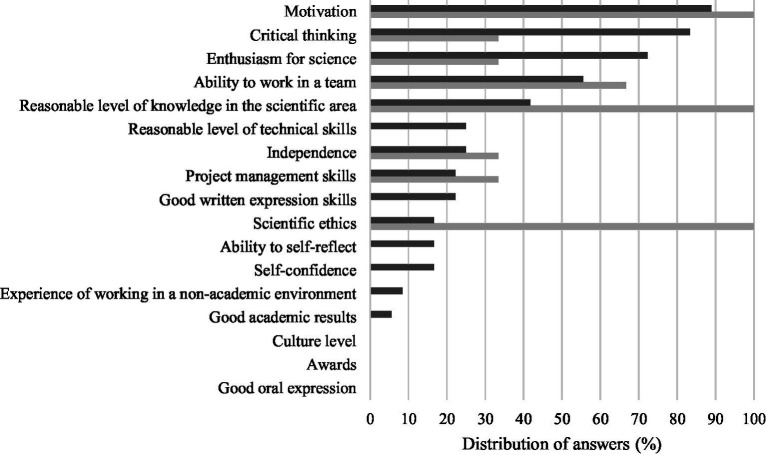
Five most important students’ attributes when starting research/training activities selected by students (dark gray) and supervisors (light gray).

Among the multiple students’ attributes presented to the participants in the questionnaire, overall, the five attributes most frequently selected by students as the utmost important in the beginning of the research/training activities were motivation (88.9%), critical thinking (83.3%), enthusiasm for science (72.2%), ability to work in a team (55.6%) and reasonable level of knowledge in the scientific area (41.7%) (Figure 1). For supervisors, when starting, the most appreciated students’ attributes were considered to be motivation (100%), reasonable level of knowledge in the scientific area (100%), scientific ethics (100%), ability to work in a team (66.7%), enthusiasm for their research field (33.3%), independence (33.3%), critical thinking (33.3%) and project management skills (33.3%) (Figure 1).

**Figure 2:**
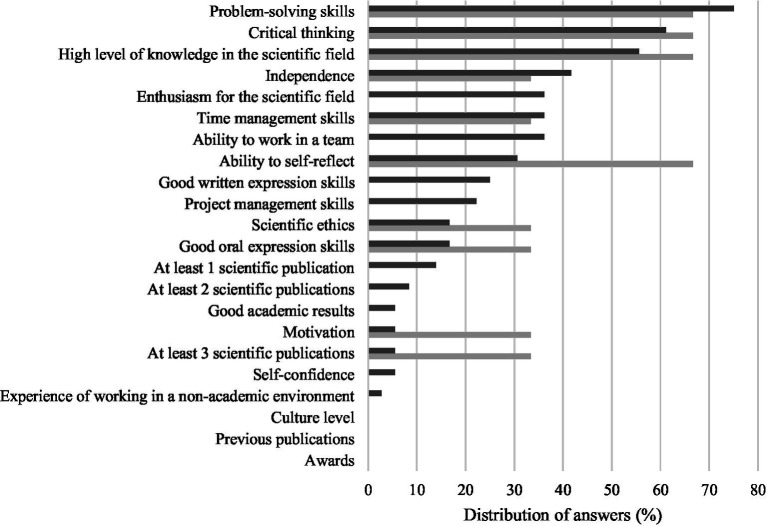
Five most important students’ outcomes at the end of the research/training period selected by students (dark gray) and supervisors (light gray).

When considering the end of the research activities, for students the most important outcomes of the research/training period were problem-solving skills (75%), critical thinking (61.1%), high level of knowledge in the scientific area (55.6%), independence (41.7%), and enthusiasm for their research field, time management skills and ability to work in a team in an equal manner (36.1%), (Figure 2). From the supervisors’ point of view, high level of knowledge in the scientific area, critical thinking, problem-solving skills and ability to self-reflect were equally selected as the most consensual expected outcomes (66.7% each) (Figure 2).

**Figure 3:**
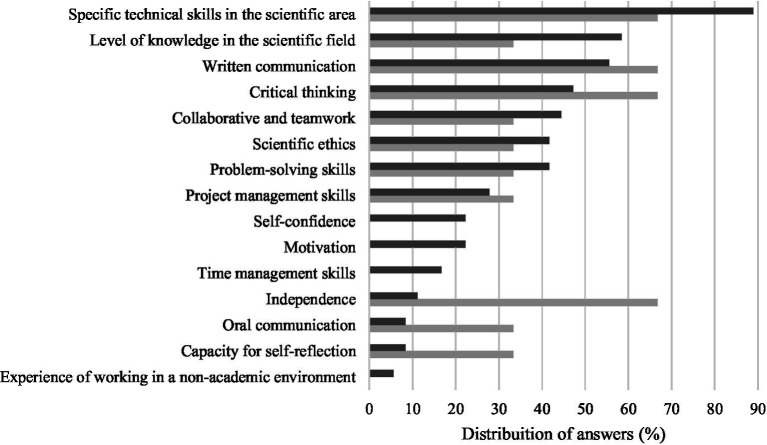
Five students’ attributes that the supervisor has the greatest responsibility for helping to develop selected by students (dark gray) and supervisors (light gray).

When asked to point out which were the attributes that students expected to develop the most with the aid of their supervisors, the answers included specific technical competences in their scientific area (88.9%), level of knowledge acquired in their scientific area (58.3%), written communication (55.6%), critical thinking (47.2%) and collaborative and teamwork skills (44.4%) (Figure 3). On the other hand, supervisors indicated the development of specific technical competences in their scientific area (66.7%) and written communication (66.7%), critical thinking (66.7%), independence (66.7%), as the major students’ attributes dependent on their guidance (Figure 3).

**Figure 4:**
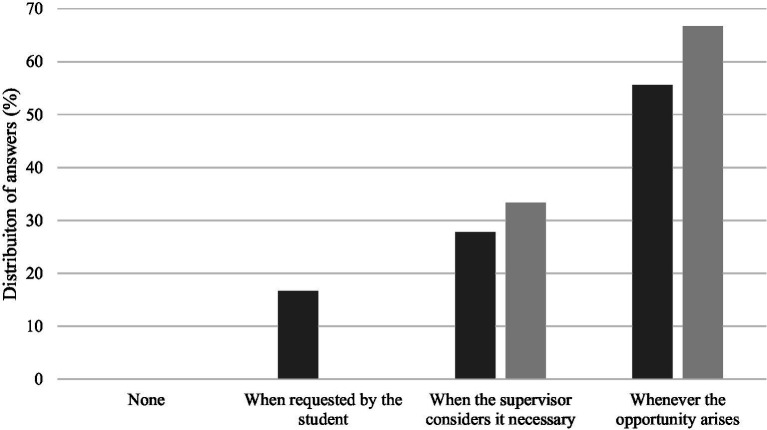
Level of guidance expected to receive/provided during the research/training period in the opinion of students (dark gray) and supervisors (light gray).

Most of the inquired students expected to receive guidance at every opportunity (55.6%) or whenever the supervisor considered it necessary (27.8%) and few expected guidance only when asked (16.7%) (Figure 4). The perception of the level guidance provided by the supervisors was mostly at every opportunity (66.7%) or whenever the supervisor thought it was necessary (33.3%) (Figure 4).

### 3.2 Mental health impact

**Figure 5:**
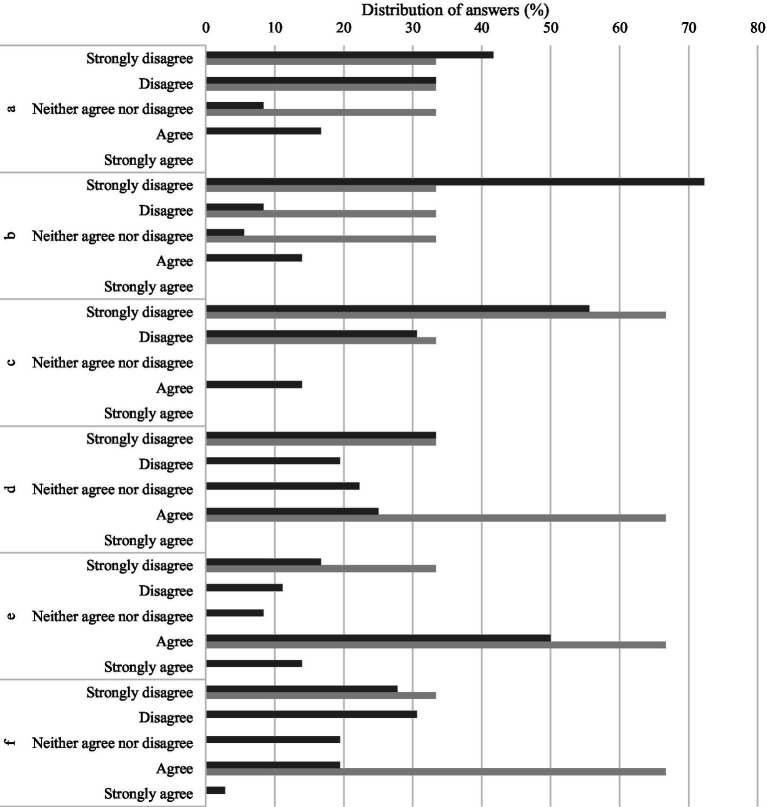
Impact of the experiences lived by students through the research/training period, in the opinion of students (dark gray) and supervisors (light gray). **(A)** The supervision experience had a negative impact on my mental health. **(B)** The relationship with my supervisor experience had a negative impact on my mental health. **(C)** The research group environment had a negative impact on my mental health. **(D)** The progress of the research project/training program had a negative impact on my mental health. **(E)** My personal expectations had a negative impact on my mental health. **(F)** The expectations of my supervisor had a negative impact on my mental health.

The majority of students either strongly disagreed (41.7%) or disagreed (33.3%) with the idea that the supervision experienced had a negative impact on their mental health, and three neither agreed nor disagreed with this statement (8.3%) (Figure 5A). Nonetheless, six students (16.7%), all from post-graduation study programs (1 PhD student, 4 MSc students and 1 student from a Veterinary Specialization program—data not shown), agreed with the statement. From the supervisors’ perception, none thought that the experience had a negative toll on their students (Figure 5A).

When the focus was on the relationship with the supervisor, most students (72.2%) strongly disagreed that it caused a negative impact on their mental health, but five students (13.9%), again all from post-graduation study programs (1 PhD student, 3 MSc students and 1 student from a Veterinary Specialization program – data not shown), confirmed that indeed it was impacting them negatively (Figure 5B). As before, none of the supervisors thought that the relationship with their students had a negative impact on them (Figure 5B).

Only five students (13.9%) reported that the research group environment was affecting them (4 MSc students and 1 student from a Veterinary Specialization program—data not shown). The remaining students either disagreed (30.6%) or strongly disagreed (55.5%) with the idea (Figure 5C). The supervisors either disagreed (33.3%) or strongly disagreed (66.7%) with the statement (Figure 5C).

Though about half of the students did not consider that the progress of the research project/training program was affecting them, 25% agreed that it had a negative impact on their mental health (Figure 5D). The answers from the supervisors were divergent, with one strongly disagreeing and the remaining two agreeing with a possible negative on students’ mental impact (Figure 5D).

The impact of personal expectations on students’ mental health was also questioned, and the majority of students confirmed it, either agreeing (50%) or strongly agreeing (13.9%) that their own expectations may impact their mental wellbeing (Figure 5E). Again, the perceptions of the supervisors were opposing, with two agreeing and the other one strongly disagreeing with the statement (Figure 5E).

Looking to the other side of the student-supervisor relationship, the impacts of the supervisor’s expectations were also analyzed. More than half of students claimed that their supervisor’s expectations did not have a negative impact on their mental health. Nevertheless, seven students (19.4%) agreed that it did impact them negatively, and one (2.8%) strongly agreed with this statement (Figure 5F). As before, the supervisors’ perceptions were not fully aligned, with two agreeing and the other one strongly disagreeing with the statement (Figure 5F).

### 3.3 Supervision quality and future academic career

**Figure 6:**
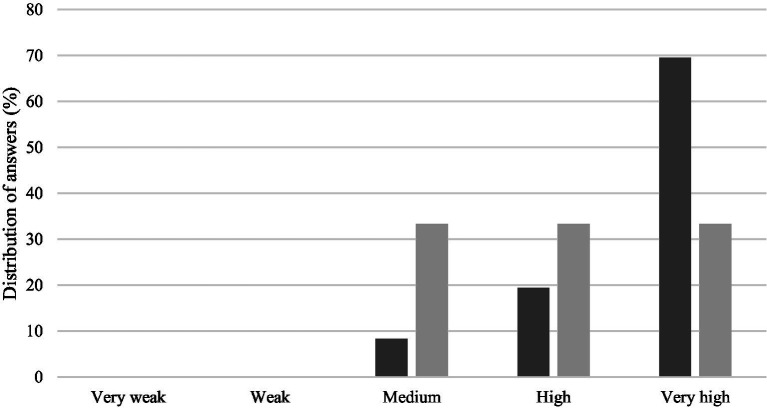
Quality of the supervision received/provided in the view of students (dark gray) and supervisors (light gray).

Students reported that generally the quality of the provided supervision was very high (66.7%) (Figure 6). Each supervisor, in a self-evaluation manner, either classified their own guidance quality as medium, high or very high.

**Figure 7:**
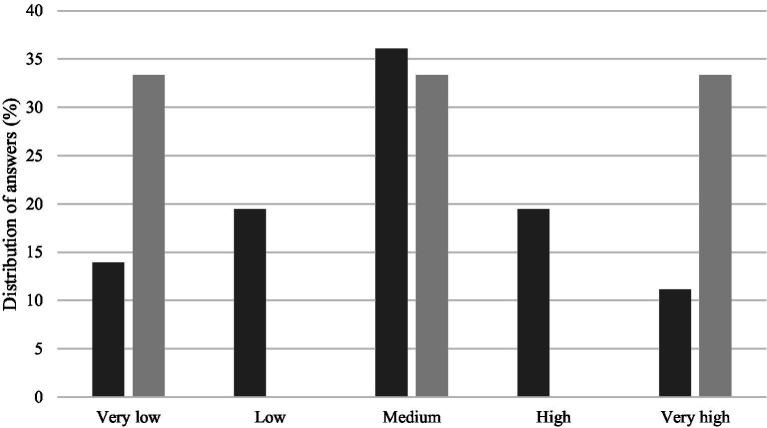
Probability of following a career in an academic environment, in the opinion of students (dark gray) and supervisors (light gray).

The final activity of the questionnaire was to classify the likelihood of each participant pursuing a career in an academic environment. The answers were divided into three almost evenly distributed groups: a group of the students that classified that possibility as very low (13.9%) or low (19.4%), a group that considered it of being of a medium likelihood (36.1%), and the group which classified that probability as high (19.4%) or very high (11.2%) (Figure 7). The opinions of the supervisors inquired on the likelihood of their students having an academic career was divided between of being of very low, medium and very high probability (Figure 7).

## 4 Discussion

The present study focused on the perceptions and expectations of students from a Portuguese Veterinary Medicine School and their supervisors on students’ attributes, the quality of supervision, and on the impact of the supervision process on students’ mental health. This study was performed as a starting point of a continuous procedure to be established to better understand the students’ point of view, allowing to adapt and improve the supervision process.

Previous to the in-depth discussion of the results, the distribution of the student participants in each education program (bachelor’s, master’s, doctorate, veterinary specialization) should be clarified. The Faculty of Veterinary Medicine of the University of Lisbon, Portugal, collaborates with other institutions of Higher Education, both at international and national levels, allowing students from these institutions to participate in the education and research activities developed there. Though not all students that participated in the present study were Veterinary Medicine students, as would be expected, the majority indeed were (24/36). It is also important to mention that, due to the changes introduced by the Bologna higher education reform process in Europe, in order to complete the Veterinary Medicine education program, after finishing the core curriculum, all students must undertake a final master’s dissertation succeeding a previous clinical/technical/research training period.

### 4.1 Students’ attributes

Our results show that there was a partial overlapping between students’ and supervisors’ opinions on what are the most important students’ attributes/outcomes. It was very interesting to observe that, at least in some cases, students and supervisors shared a common view on what are the student attributes expected, as well as on the results after the supervision process. While “motivation” and “ability to work in a team” were selected as valuable at the beginning of the research/training period by both students and supervisors in a similar frequency, some students perceived other attributes as valuable, though none of the supervisors did (e.g., “reasonable level of technical skills” or “good written expression skills”). These findings seem to point out the preconceived vision of the ideal research student. The answers given by the student participants might in fact reflect what they thought to be their supervisors’ expectations. As previously observed, PhD students try to adapt themselves to the reality of their academic context and act accordingly to accomplish their studies (7). The same could also be assumed for undergraduate and master’s research students. In the doctoral study program, which is the most studied supervision model, the selection of new students is described as being complicated, as both the needs and abilities of students and supervisors must be considered, and that students with little research experience might not be fully aware of the endeavors associated with such research programs (8). In a study focusing on the desired features of doctoral students’ to be enrolled on highly ranked doctorate-granting neuroscience programs in the United States, the most commonly reported selection criteria included their skills for basic research, written and oral communication, bench skills, critical thinking, internal motivation, ethical behavior and work ethic (9). Remarkably, all supervisors considered “scientific ethics” as an important student attribute, contrasting with less than 20% of the participating students. A previous study reported that students’ perception on the implication of research ethics was positively correlated with the level of studies (10). In fact, in our study the only students which selected “scientific ethics” were from the master’s and doctoral programs (data not shown).

Regarding the end of the research/training period, a similar scenario was observed. Most supervisors expected that their supervisees would be able to gain the “ability to self-reflect,” but only less than one third of students did perceive it as important. On the other hand, some students considered as an important outcome “enthusiasm for the scientific field” or the “ability to work in a team,” while none of the supervisors did. While doctoral graduates are associated with broad range of attributes that go beyond specific knowledge and research skills, including communication, organizational, inter and intrapersonal skills (11), the same is not exactly true for undergraduate research students and master’s graduates.

Moreover, in the specific setting of our study, most of the master’s students were from the Integrated Master in Veterinary Medicine, who must perform their final research project in a field which they consider to be useful for their future professional life. According to van der Marel, in professionally oriented graduation research projects, the main focus is in developing professional competencies over research competencies (12), which might also be the view of some of the students that participated in our study.

Concerning the attributes which the supervisor has the greatest responsibility to help developing, despite convergence between students and supervisors in the selection of some attributes, supervisors tended to select “independence” or “oral communication” as relevant rather than “self-confidence” or “motivation.” Curiously, the selection of the attributes “self-confidence” and “motivation” by some of the students may indicate an expectation for a more supportive type of supervision. On the other hand, the choices of the supervisors pointed toward a perceived greater responsibility of assuring that their supervisees gained their own autonomy or the skills required to move forward in academia or to undertake further research activities. This gain of autonomy assumes even further significance when considering the students enrolled in veterinary specialization and doctoral programs. These programs are commonly based on a minimum training period of 3-years, designed to provide students with specific education and practices in a selected scientific area. Like for academic degrees, veterinary specialization students must be supervised, in this case by an internationally recognized Specialist, and complete a final evaluation step, in the form of an exam, before being granted with the title of Specialist. Though this title is not granted by higher education institutions, many of the Specialists which supervise these programs are also in academia, and therefore it is plausible that these supervisors assume the same type of supervision and expectations for students from both postgraduate research and veterinary specialization programs. Moreover, at the doctoral study level, it is suggested that supervisors need to promote the development of students’ academic independence, along with collaboration skills, since this will facilitate their journey along the study program (2).

### 4.2 Type of guidance and mental health impact

In this study, the majority of students reported that they expect to receive guidance either whenever the opportunity arises or when the supervisor considers it necessary. This indicates that the inquired students were relying on the active role of their supervisors for guidance throughout the research/training period. In fact, the study by Orawczyk (13) also underscored the importance of a structured learning to enhance supervision effectiveness. These findings are not surprising, since most of the participants are undergraduate and master’s students, thus lacking academic independence. Nevertheless, doctoral and veterinary specialization students also need guidance, though not exclusively in scientific or academic matters. Indeed, the work of Varghese (14) highlighted how supervisory leadership styles may directly influence supervisee outcomes and Ädel et al. (15) noted that doctoral supervision often involves personal commitment (e.g., by a mentor or supporter), whereas undergraduate supervision is typically institutional (e.g., by an expert or project manager).

Additionally, in this study, six 5-point Likert scale questions were presented to the participants to assess their perception on the impact of the supervision process on students’ mental health. Overall, students seemed not to be seriously affected by the supervision process, by the relationship with the supervisor or even by the research group environment. Our results show that, while some students reported that the progress of the research or training affected their mental wellbeing, they also considered that their own expectations influenced their mental health status, as well as their supervisors’ expectations, to a lesser extent. Moreover, a similar perception was reported by the supervisors.

Previous studies have reported situations of anxiety and other mental health issues, experienced by PhD students as a result of considering themselves the only agents responsible for the completion of their doctoral studies, and of perceiving “workaholism” and perfectionism as necessary for academic progress (16, 17). On the other hand, it is also well known that the relationship developed between supervisor and supervisee highly affects students’ wellbeing and productivity (18–20).

### 4.3 Supervision quality and future academic career

A recent study on the perceptions of supervisees and supervisors on the quality of supervision showed that informational, emotional, instrumental and co-constructional support are the main characteristics of a high-quality supervision (4). Another study on PhD student satisfaction has reported that the most relevant predictor of personal and professional satisfaction is how frequently the students meet with their supervisors, with an optimal frequency of at least one meeting per week (21). Additionally, the same study showed that the satisfaction levels regarding students’ personal and professional relationships decreased as the number of supervised students increased, pointing toward the need for establishing a necessary balance between the number of supervisees and the time availability of supervisors for frequent meetings (21). For master’s students, it has been described that effective thesis supervision is considered to rely on the supervisors’ abilities to adapt their actions and role in the supervisee-supervisor relationship according to the characteristics of the students, changing whenever necessary (22). In this study, the results of the questionnaire showed that most students considered that the supervision received was of high or very high quality, though the criteria used to evaluate the supervision were not assessed. The supervisors’ self-evaluation on the quality of the supervision provided requires, primarily, the ability to self-reflect on their own pedagogical practices, and the results obtained could have been affected by multiple factors, namely by perceived (in)experience, previous supervisions, time availability or personal expectations.

The results focusing on the students’ probability of pursuing an academic career did not show any clear pattern. As mentioned above, the specific set of students that participated in the present study might have influenced these results. Generally, undergraduate students have limited experience in higher education and are still clarifying their interests and professional goals. In our experience, most Veterinary Medicine students are focused on clinical practice as their primary choice of professional career and master’s thesis project. In a similar fashion, Veterinary Specialization students are mainly engaged in deepening their technical and scientific expertise in their field of choice, with the objective of enhancing and gaining new professional skills. Furthermore, it is the group of doctoral students that is commonly viewed as the next generation of academics, according to the Humboltdian tradition. Nevertheless, multiple factors might discourage doctoral students from continuing an academic path, including institutional norms, career attractiveness, academic work besides research, publishing expectation and low remuneration (23–25).

### 4.4 Study limitations

The present study intended to assess the perceptions, expectations and mental health implications of the supervision process in our particular pedagogical context. The students which participated in the survey are representative of the reality observed in the Faculty of Veterinary Medicine of the University of Lisbon, though for additional insights, namely for comparisons between groups, a larger number of participants and a more balanced sample would have been beneficial. Likewise, the number of supervisors which answered the questionnaire was limited, which might not represent fully the natural occurring variability within this higher education institute. Moreover, the questionnaire, though bringing very valid information, was not prepared for an in-depth examination of the reasons behind some of the results which it produced. Considering this, our study suggests that a larger and more homogeneous student sample, along with the inclusion of a greater number of supervisors, should be taken into consideration in the design of a future study.

## 5 Conclusion

Our results show that research/veterinary specialization students and their supervisors share some of the same expectations regarding the most important student features in the beginning of the research/training process, as well as the most important results of the supervision process. Students expect supervisors to help develop mainly technical skills, while, besides technical skills, the supervisors feel responsible for helping develop student autonomy. It is advisable for students and supervisors to express and discuss their expectations before the beginning of the study / training programs, in order to identify misalignments which can later damage the student-supervisor relationship or the students’ mental health. The students participating in this study expected an active engagement of the supervisors, and expressed that the factors most affecting their mental health were the progress of their research or training program and their own personal expectations. The supervisors also perceived that their expectations induced a negative impact on students’ mental health. Though most students evaluated the supervision received as of high or very high quality, the supervisors’ perception on this issue was not uniform. Regarding the possibility of following an academic career, both students’ and supervisors’ answers were diverse, with no clear agreement on the probability of the research/specialization students pursuing an academic career in the future.

Further and continuous research is needed to better understand both the needs and expectations of students and supervisors, especially in Veterinary Medicine Schools, to help implement a supportive structure for students undergoing research or training intensive programs in this scientific area, and also to help design further pedagogical training courses for supervisors according to the major issues identified.

## Data Availability

The datasets used and/or analyzed in the current study are available from the corresponding author upon reasonable request.
